# Association of Antibiotics and Other Drugs with Clinical Outcomes in Metastatic Melanoma Patients Treated with Immunotherapy

**DOI:** 10.1155/2021/9120162

**Published:** 2021-07-23

**Authors:** Manish D. Angrish, Arun Agha, Rossanna C. Pezo

**Affiliations:** ^1^Biological Sciences Platform, Sunnybrook Research Institute, Toronto, Canada; ^2^Department of Medicine, University of Toronto, Toronto, Ontario, Canada; ^3^Division of Medical Oncology, Sunnybrook Health Sciences Centre, Toronto, Ontario, Canada

## Abstract

Immune checkpoint inhibitors (ICIs) targeting the programmed cell death protein-1 (PD-1) and programmed cell death ligand-1 (PD-L1) have improved survival in many advanced cancers including advanced melanoma, renal cell, urothelial, and non-small-cell lung cancers. However, not all patients respond, and immune-related adverse events (irAEs) are common. Commensal gut bacteria may serve as an immunoregulatory link-mediating ICI response and toxicity. Recent studies have shown that a lack of bacterial diversity, known as gut dysbiosis, can have an adverse impact on patients' response to ICIs and predispose to the development of irAEs. Data were collected from 167 patients with metastatic melanoma who received antibiotics within 30 days prior to and/or after initiation of ICI and patients who received NSAIDs, statins, steroids, or proton-pump inhibitors (PPI) within 30 days prior to ICI initiation. The primary outcome was time-to-discontinuation (TTD) of ICI therapy, measured from the date of ICI initiation to the last treatment date. The secondary outcome of interest was toxicity, with incidence of irAEs graded as per the Common Terminology Criteria for Adverse Events (CTCAE), version 5.0. Here, we demonstrate that individuals who received antibiotics had a significantly shorter time-to-discontinuation (TTD) of the ICI therapy as opposed those who were not administered antibiotics. Consistent with results from previous research, we propose that antibiotics have a negative effect on a patient's response to ICI therapy, most likely due to the result of gut dysbiosis, and should be critically assessed in terms of their use in patients undergoing ICI treatment.

## 1. Introduction

Immune checkpoint inhibitors (ICIs) targeting programmed cell death protein-1 (PD-1) and cytotoxic T-lymphocyte-associated protein 4 (CTLA-4) are used in the treatment of multiple solid tumors. Recent studies have observed a link between gut bacterial diversity and ICI responses and toxicities. The types of bacteria present within gut are altered by antibiotics, which eliminate certain commensal bacterial species and lead to overgrowth of others, decreasing gut bacterial diversity. Poor bacterial diversity is thought to modify host metabolic capacity, creating a condition known as “dysbiosis” [1]. Although not fully understood, gut microbiota in a dysbiosis state is thought to undergo changes in immune signaling that may lead to reduced ICI efficacy [1].

Previous studies have identified a potential link between antibiotic use and inferior clinical outcomes on ICIs; less is known about the impact of other common supportive medications. Therefore, we sought to examine the impact of antibiotics and other drugs on clinical outcomes in metastatic melanoma patients receiving ICIs.

## 2. Materials and Methods

A single-centre retrospective chart review was performed according to CONSORT criteria (Supplementary Figure 1) using electronic medical records to identify patients ≥18 years with a diagnosis of metastatic melanoma receiving first-line ICIs (PD-1 inhibitors alone or in combination with CTLA-4 inhibitors) between 2012 and 2018. Patients receiving single-agent CTLA-4 inhibitors were excluded. The study was approved by the Sunnybrook Health Sciences Research Ethics Board.

Included patients received either antibiotics within 30 days prior to and/or after initiation of ICI, or NSAIDs, statins, steroids, or PPIs within 30 days prior to ICI initiation and were compared to a cohort of patients who did not receive these medications during the same study period. Primary outcome was time-to-treatment discontinuation (TTD) of first-line ICI, measured from initiation to discontinuation date. TTD was used as a proxy of real-world treatment efficacy. Censored date was January 1, 2020. Secondary outcome of interest was the incidence of irAEs, graded as per CTCAE v5.0.

Baseline demographics between groups were compared using Pearson's chi-squared test for categorical data, and *t*-test for continuous variables. For analysis of primary and secondary outcomes, Kaplan–Meier survival curves and Pearson's chi-squared were used, respectively.

## 3. Results

**Figure 1:**
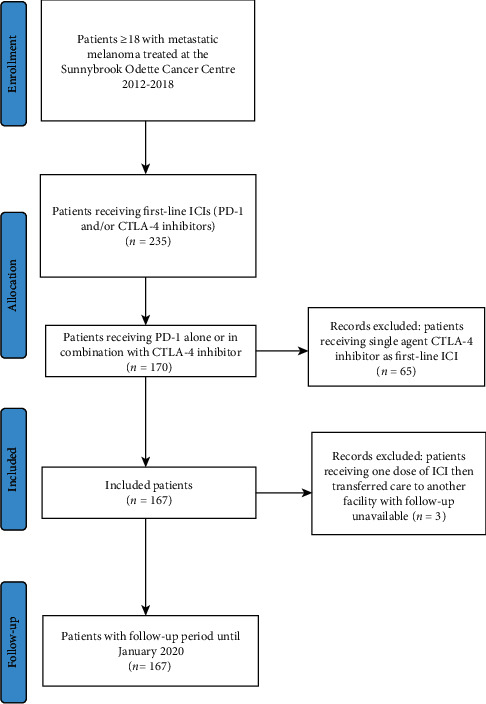
PRISMA flow diagram for patients included in the retrospective cohort analysis.

Of 235 patients with metastatic melanoma receiving treatment with ICIs at our institution, a total of 167 patients receiving either PD-1 inhibitor alone (pembrolizumab or nivolumab) or in combination with the CTLA-4 inhibitor ipilimumab were included (Figure 1). Median (range) age was 62 (18–95) years at the first dose of ICI, and 63% of study participants were male (Table 1). The majority received single-agent pembrolizumab, and use of combination ipilimumab and nivolumab was low due to limited accessibility and funding in Ontario at the time. A total of 72 patients (43.1%) discontinued treatment due to progressive disease, and 37 (22.2%) discontinued due to toxicity.

NSAIDs (*n* = 114) were the most commonly prescribed medications, followed by steroids (*n* = 72), statins (*n* = 65), PPIs (*n* = 63), and antibiotics (*n* = 34). Patients receiving steroids prior to ICI initiation had a significantly higher number of irAEs compared to patients not receiving steroids (46/72 vs. 42/95 patients, respectively (*p*=0.013). There were no significant differences in irAEs for patients taking other medications: antibiotics, *p*=0.34; NSAIDs, *p*=0.74; statins, *p*=0.635; PPIs, *p*=0.26.

**Figure 2:**
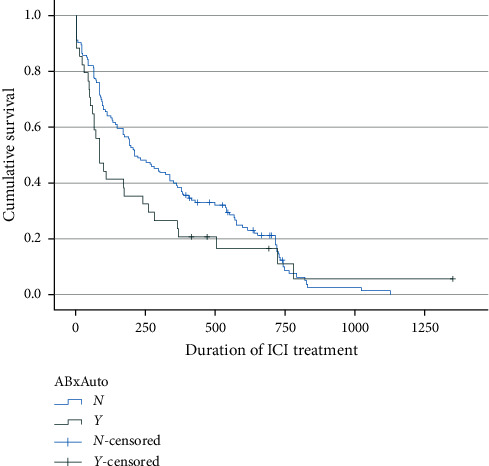
Use of antibiotics and duration of ICI treatment.

A significant reduction in TTD was observed for patients receiving antibiotics vs. those not receiving antibiotics (Table 2). Of 34 patients who received antibiotics, 30 discontinued treatment (TTD = 260.5 days), whereas 120 of the 133 patients who did not receive antibiotics discontinued treatment (TTD = 332.4 days (*p*=0.03), as shown in Figure 2. None of the other concomitant medications had a significant impact on overall TTD (NSAIDs, TTD = 310.2 vs. 331.9 days; *p*=0.97; statins, TTD = 282.4 vs. 339.6 days; *p*=0.54; PPIs, TTD = 266.1 vs. 346.8 days, *p*=0.09; and steroids, TTD = 285.9 vs. 339.4 days; *p*=0.19).

## 4. Discussion

This study provides further support for a detrimental effect of antibiotics on the efficacy of ICIs. However, we were not able to find a negative impact of other concomitant medication use on ICI efficacy. We observed greater irAEs in patients receiving steroids prior to ICI initiation. Although the reasons for steroid use prior to ICIs were unknown, steroids are often used as supportive medications in oncology patients, especially in those with brain metastases and to treat various symptoms such as pain, fatigue, and poor appetite. There was no significant difference in irAEs in patients using vs. not using other concomitant medications (antibiotics, PPIs, NSAIDs, and statins).

This higher incidence of irAEs in patients receiving steroids prior to ICI therapy may be due to a negative impact of these drugs on diversity of gut microbiota [2]. A recent study in advanced NSCLC patients reported significantly inferior objective RR, PFS, and/or OS in patients taking ≥10 mg/day of prednisone versus those taking lower doses [3]. However, the negative impact of steroids may be due to their use in patients with poorer performance status, as a large retrospective study showed that patients taking baseline steroids were more likely to have poorer ECOG scores at baseline and more likely to have brain and/or liver metastases at diagnosis [4]. A majority of patients in our study had ECOG scores of 0 or 1.

A key finding to highlight in our study was our observation of a shorter TTD for patients who received antibiotics. This is important as many patients are often prescribed broad-spectrum antibiotics, which eliminate larger numbers of commensal bacteria, for routine mild illnesses where their use may not necessarily be warranted. However, we were not able to link the use of specific classes of antibiotics or drug doses to shorter TTD, as this information was not commonly available in our retrospective dataset. In a study conducted by Dethlefsen et al., receipt of the antibiotic ciprofloxacin led to disruption of 30% of the gut microbiota, resulting in loss of bacterial diversity [5]. In a recent study by Cortellini et al., cancer patients administered steroids, PPIs, and systemic antibiotics had significantly worse clinical outcomes on PD-1/PD-L1 inhibitors, presumably due to their detrimental effect on the immune system [6]. Kuczma et al. observed that antibiotic prophylaxis of mice treated with cyclophosphamide reduced antitumor T-cell responses, as well as led to a diminished effect of adoptive T-cell therapy, with decreased tumor-specific CD4+ T-cells [7].

Pinato et al. examined the effect of administering antibiotics prior to (pATB) and concurrently with (cATB) ICIs. Although cATB did not significantly impact efficacy, pATB patients had significantly worse overall survival (*p* < 0.01) compared to non-pATB patients (NSCLC (2.5 vs. 26 months), melanoma (3.9 vs. 14 months), and other tumor types (1.1 vs. 11 months)) [8]. Our findings are also consistent with a study by Derosa et al. which found a significant increase in primary disease progression, and a significant decrease in PFS and OS for advanced renal cell and NSCLC patients who received antibiotics within 30 days prior to ICIs, as opposed to those who did not [9].

In addition to observing a correlation between antibiotic use and a decreased response to ICIs, Routy et al. found that the transplantation of fecal microbiota from an ICI-responder patient into germ-free or antibiotic-treated mice improved response to ICIs [10]. Hakozaki et al. compared fecal contents of patients receiving vs. not receiving pre-ICI antibiotics and demonstrated an adverse impact of antibiotics on both gut bacterial diversity and ICI responses [11].

## 5. Conclusions

Our results are in agreement with prior studies observing a detrimental effect of antibiotics on ICI responses. The potential negative impact of antibiotics and other commonly prescribed medications suggest that it is important to carefully consider risks and benefits of such medication use in patients receiving ICIs. Future work should examine whether factors such as drug dose and timing of concomitant medication administration play a role in ICI efficacy and toxicity.

## Figures and Tables

**Table 1:** Patient characteristics.

Characteristics	Number of patients (*n* = 167), no. (%)
*Age*	
Mean	66.5
Median	68.0
Range	18–95

*Sex*	
Male	105 (62.9)
Female	62 (37.1)

*Performance status (ECOG score)*	
0	143 (85.6)
1	16 (9.6)
2	6 (3.6)
3	1 (0.6)
4	1 (0.6)

*Subtype*	
Cutaneous	122 (73.1)
Desmoplastic	1 (0.6)
Mucosal	7 (4.2)
Nevoid melanoma	1 (0.6)
Nodular	28 (16.8)
Pigment synthesizing melanoma	1 (0.6)
Spindle cell malignant melanoma	2 (1.2)
Not known	5 (3.0)

*BRAF V600 mutation*	
No	105 (62.9)
Not known	8 (4.8)
Yes	54 (32.3)

*Sites of metastasis*	
Bone	49 (29.3)
Central nervous system	56 (33.5)
Lymph nodes	143 (85.6)
Liver	73 (43.7)
Lung	119 (71.3)
Soft tissue and others	108 (64.7)

*ICI treatment received*	
Ipilimumab + nivolumab	6 (3.6)
Nivolumab	19 (11.4)
Pembrolizumab	142 (85.0)

*Immune-related adverse events*	
Gastrointestinal	33 (9.8)
Pneumonitis	16 (9.6)
Thyroid	14 (8.4)
Diabetes	2 (1.2)
Adrenal insufficiency	5 (3.0)
Hypophysitis	2 (1.2)
Neurologic	12 (7.2)
Hematologic	1 (0.6)
Renal	9 (5.4)
Musculoskeletal	11 (6.6)
Skin	35 (21.0)

*Cause of ICI discontinuation (if discontinued)*	
Progressive disease	72 (43.1)
Toxicity	37 (22.2)
Best response	24 (14.4)
Ongoing	15 (9.0)
Death	9 (5.4)
Not known	8 (4.8)
Others	2 (1.2)

**Table 2:** Comparison of the time-to-discontinuation (TTD) in days among patients who received concomitant medications (CI = 95%).

Medication received	Mean	Std. deviation	Lower bound	Upper bound
*Antibiotics*				
No	332.4	26.2	281.2	383.7
Yes	260.5	63.4	136.2	384.8

*NSAIDs*				
No	331.9	45.7	242.3	421.5
Yes	310.2	28.8	253.8	366.6

*Statins*				
No	339.6	33.2	274.5	404.7
Yes	282.4	34.2	215.4	349.4

*PPI*				
No	346.8	31.1	285.8	407.8
Yes	266.1	37.8	191.9	340.2

*Steroids*				
No	339.4	31.1	278.5	400.3
Yes	285.9	38.0	211.4	360.5

NSAIDs, nonsteroidal anti-inflammatory drugs; PPI, proton-pump inhibitor.

## Data Availability

The data used to support the findings of this study are available from the corresponding author upon request.

## Supplementary Materials

Supplementary Materials Supplementary Figure 1: STROBE checklist for observational studies.

## References

[1] Lange K., Buerger M., Stallmach A., Bruns T. (2016). Effects of antibiotics on gut microbiota. *Digestive Diseases*.

[2] Tetel M. J., De Vries G. J., Melcangi R. C., Panzica G., O’Mahony S. M. (2018). Steroids, stress and the gut microbiome-brain axis. *Journal of Neuroendocrinology*.

[3] Arbour K. C., Mezquita L., Long N. (2018). Impact of baseline steroids on efficacy of programmed cell death-1 and programmed death-ligand 1 blockade in patients with non-small-cell lung cancer. *Journal of Clinical Oncology*.

[4] Drakaki A. (2019). Association of systemic corticosteroids with overall survival in patients receiving cancer immunotherapy for advanced melanoma, non-small cell lung cancer or urothelial cancer in routine clinical practice. *Annals of Oncology*.

[5] Dethlefsen L., Huse S., Sogin M. L., Relman D. A. (2008). The pervasive effects of an antibiotic on the human gut microbiota, as revealed by deep 16S rRNA sequencing. *PLoS Biology*.

[6] Cortellini A., Tucci M., Adamo V. (2020). Integrated analysis of concomitant medications and oncological outcomes from PD-1/PD-L1 checkpoint inhibitors in clinical practice. *Journal for Immunotherapy of Cancer*.

[7] Kuczma M. P., Ding Z.-C., Li T. (2017). The impact of antibiotic usage on the efficacy of chemoimmunotherapy is contingent on the source of tumor-reactive T cells. *Oncotarget*.

[8] Pinato D. J., Howlett S., Ottaviani D. (2019). Association of prior antibiotic treatment with survival and response to immune checkpoint inhibitor therapy in patients with cancer. *JAMA Oncology*.

[9] Derosa L., Hellmann M. D., Spaziano M. (2018). Negative association of antibiotics on clinical activity of immune checkpoint inhibitors in patients with advanced renal cell and non-small-cell lung cancer. *Annals of Oncology*.

[10] Routy B., Emmanuelle L. C., Lisa D. (2019). Gut microbiome influences efficacy of PD-1–based immunotherapy against epithelial tumors. *American Association for The Advancement of Science*.

[11] Hakozaki T., Richard C., Elkrief A. (2020). The gut microbiome associates with immune checkpoint inhibition outcomes in patients with advanced non-small cell lung cancer. *Cancer Immunology Research*.

